# Susceptibility Testing Is Key for the Success of Cefiderocol Treatment: A Retrospective Cohort Study

**DOI:** 10.3390/microorganisms9020282

**Published:** 2021-01-30

**Authors:** Alexandre Bleibtreu, Laurent Dortet, Remy A. Bonnin, Benjamin Wyplosz, Sophie-Caroline Sacleux, Liliana Mihaila, Hervé Dupont, Helga Junot, Vincent Bunel, Nathalie Grall, Keyvan Razazi, Clara Duran, Pierre Tattevin, Aurélien Dinh, on behalf of the Cefiderocol French Study Group

**Affiliations:** 1 Infectious Disease Unit, La Pitié-Salpétrière University Hospital, AP-HP, University of Paris, 75013 Paris, France; alexandre.bleibtreu@aphp.fr; 2 Microbiology Laboratory, Bicêtre University Hospital, AP-HP Paris Saclay University, 94270 Le Kremlin-Bicêtre, France; laurent.dortet@aphp.fr (L.D.); remy.bonnin@u-psud.fr (R.A.B.); liliana.mihaila@aphp.fr (L.M.); 3 Associate French National Center for Antimicrobial Resistance, 94275 Le Kremlin-Bicêtre, France; 4 Infectious Disease Unit, Bicêtre University Hospital, AP-HP Paris Saclay University, 94270 Le Kremlin-Bicêtre, France; benjamin.wyplosz@aphp.fr; 5 Intensive Care Unit, Paul Brousse Hospital, AP-HP Paris Saclay University, 94800 Villejuif, France; 6 Intensive Care Unit, Amiens University Hospital, 80054 Amiens, France; dupont.herve@chu-amiens.fr; 7 Pharmacy Department, La Pitié-Salpétrière University Hospital, AP-HP, University of Paris, 75013 Paris, France; helga.junot@aphp.fr; 8 Pneumology Department, Bichât University Hospital, AP-HP, University of Paris, 75018 Paris, France; vincent.bunel@aphp.fr; 9 IAME, INSERM, University of Paris, 75870 Paris, France; nathalie.grall@aphp.fr; 10 Microbiology Laboratory, Bichât University Hospital, AP-HP, University of Paris, 75018 Paris, France; 11 Intensive Care Unit, Henri Mondor University Hospital, AP-HP, 94010 Créteil, France; keyvan.razazi@aphp.fr; 12 Infectious Disease Unit, Raymond-Poincaré University Hospital, AP-HP Paris Saclay University, 92380 Garches France; clara.drn@gmail.com; 13 Infectious Disease and Intensive Care Unit, Pontchaillou University Hospital, 35000 Rennes, France; pierre.tattevin@chu-rennes.fr

**Keywords:** cefiderocol, bacterial resistance, carbapenem, respiratory tract infection, *Pseudomonas aeruginosa*

## Abstract

Cefiderocol is a novel siderophore cephalosporin, which has proven in vitro activity against carbapenem-resistant (CR) Gram-negative pathogens and stability towards all carbapenemases. The aim of this study was to describe the first cases of prescriptions and the efficacy of cefiderocol for compassionate use in the 2 months following its access in France. We performed a national retrospective study of all patients who received at least one dose of cefiderocol from 2 November 2018 to 5 November 2019. We collected clinical characteristics and outcome through a standard questionnaire. Bacterial isolates from 12 patients were centralized and analyzed in the French National Reference Center for Antimicrobial Resistance, and sequenced using Illumina technology. Finally, 13 patients from 7 French university hospitals were included in the study. The main type of infection treated by cefiderocol was respiratory tract infections (RTI, *n* = 10). The targeted bacteria were *Pseudomonas aeruginosa* (*n* = 12), including carbapenemase-producing *P. aeruginosa* (*n* = 9), *Acinetobacter baumannii* (*n* = 2), *Klebsiella pneumoniae* (*n* = 1), and *Enterobacter hormaechei* (*n* = 1). Overall, of the 12 patients whose samples were analyzed, 5 *P. aeruginosa* strains were not susceptible to cefiderocol (4 categorized as resistant and 1 as intermediate) according to Clinical and Laboratory Standards Institute (CLSI) breakpoints. If considering susceptible strains, the cure rate was 6/7, while being 0/5 among not-susceptible strains. This study underlines the necessity to test strains in adequate conditions.

## 1. Introduction

Cefiderocol is a novel siderophore cephalosporin, with in vitro activity against multidrug-resistant (MDR) Gram-negative pathogens, and stability towards all carbapenemases, including metallo-β-lactamases (MBLs) [1]. However, few data on real-life use and the clinical efficacy of cefiderocol are available.

The objective of this study was to describe the efficacy of cefiderocol as compassionate use during the early access program in France.

## 2. Materials and Methods

We performed a national retrospective study of all adult patients who received at least one dose of cefiderocol from 2 November 2018 to 5 November 2019. Standardized questionnaires were sent to the prescribers to collect patients’ baseline characteristics, infections type and management, microbiological data, reasons for cefiderocol use, doses and duration of cefiderocol treatment, concomitant antibiotic treatments, adverse events, and outcome. The research was conducted in accordance with the Declaration of Helsinki, and national and institutional standards. Patients were informed that cefiderocol was provided within a compassionate use program, and that their clinical data could be used, after anonymization, for research purposes.

Immunosuppression was defined as asplenia, neutropenia, agammaglobulinemia, organ transplant, hematologic malignancies, HIV infection with CD4 cells count <400/mm^3^, or end-stage liver disease. Immunosuppressive treatment was defined as systemic corticosteroids with a daily dose >20 mg of prednisolone equivalent during at least 3 weeks, cancer chemotherapy, or other immunosuppressive drugs (e.g., cyclophosphamide, azathioprine, cyclosporine, etc.).

Outcome was evaluated by the investigators at the patient’s most recent visit after completion of cefiderocol treatment. Primary criteria was cure, defined as survival with no residual sign of infection, and pathogen eradication.

All bacterial isolates were centralized at the French National Reference Center for Antimicrobial Resistance (Bicêtre University Hospital, Le Kremlin-Bicêtre, France), where strain analyzing and drug susceptibility testing were performed by disk diffusion, and minimum inhibitory concentrations (MICs) were determined by broth microdilution (BMD) per the Clinical and Laboratory Standards Institute (CLSI) and European Committee on Antimicrobial Susceptibility Testing (EUCAST) guidelines [2]. Cefiderocol MICs were also determined by BMD using iron-depleted and cation-adjusted Mueller–Hinton broth (ThermoFisher, Waltham, MA, USA) as recommended by CLSI guidelines [3]. The breakpoints used were those defined by the CLSI, 2019 update. All bacterial isolates were sequenced using Illumina technology as previously described [4]. Resfinder server v3.2 (https://cge.cbs.dtu.dk/services/ResFinder/) and CARD database (https://card.mcmaster.ca) were used to identify the antimicrobial resistance genes. The MLST (MultiLocus Sequence Typing) was performed using the MLST 2.0 server (http://www.genomicepidemiology.org/).

Quantitative variables are presented as mean ± standard deviation (SD), or median and interquartile range (IQR). Qualitative variables are presented as number of occurrences and relative frequencies. All statistical analyses were performed using SPSS Statistics v.17.0 software (SPSS Inc., Chicago, IL, USA).

## 3. Results

Overall, 13 patients from 7 French university hospitals were included in the study (Table 1).

The main sites of infection were respiratory tract (*n* = 10), intra-abdominal (*n* = 2), osteo-articular (*n* = 2), skin-and-skin structure (*n* = 1), and urinary tract (*n* = 1). Among them, 4 patients had multi-site infections, and 2 patients presented concomitant bacteremia.

Among the 10 patients enrolled in the intensive care unit (ICU), the mean ± SD sequential organ failure assessment (SOFA) score was 6.9 ± 4.1, and 4 (30.8%) developed septic shock.

The most common cefiderocol regimen was 2 g tid (*n* = 6), while 3 patients with acute renal failure required a regimen of 750 mg bid (*n* = 1), or tid (*n* = 2). Cefiderocol was administered as 3- or 4-h infusions in 12 patients, and 1-h infusion in one patient.

Concomitant antibiotics targeting the same bacteria were used for 9 (69.2%) patients: colistin (*n* = 8), cyclines (*n* = 2), and levofloxacin (*n* = 1).

Only 2 severe adverse events were reported. One patient suffered from renal failure, which resolved while cefiderocol treatment was not discontinued. One patient developed thrombocytopenia, which resolved after cefiderocol treatment discontinuation.

The most common pathogens targeted were *Pseudomonas aeruginosa* (*n* = 12), including 9 carbapenemase-producers, *Acinetobacter baumannii* (*n* = 2), *Klebsiella pneumoniae* (*n* = 1), and *Enterobacter hormaechei* (*n* = 1). Results of antimicrobial susceptibility testing, MLST typing, and antibacterial resistance genes at the National Reference Center for Antimicrobial Resistance are presented in Table 1, except for one patient with a *P. aeruginosa* strain (bacterial samples no longer available). Although 5 isolates targeted were finally reclassified as non-susceptible to cefiderocol (4 categorized as resistant, and 1 as intermediate), all other *P. aeruginosa* strains were susceptible. The median MICs of cefiderocol and colistin for *P. aeruginosa* were 4.0 mg/L (IQR 1.0–16.0) and 2.0 mg/L (IQR 2.0–2.0), respectively.

Analysis of the resistomes of these isolates indicated a variety of β-lactamase encoding genes. Among acquired carbapenemases identified, the *bla*_VIM-2_ (*n* = 3), *bla*_VIM-4_ (*n* = 1) and *bla*_NDM-1_ (*n* = 1) genes were identified in *P. aeruginosa* strains. The blaOXA-48-gene was identified in one isolate of *K. pneumoniae,* whereas the *bla*_OXA-23_ gene was identified in the two isolates of *A. baumannii*. In addition to these acquired carbapenemases, these isolates also produced their natural β-lactamases, *bla*_PDC-_, *bla*_ADC-_ and *bla*_ACT_-like corresponding to class C β-lactamase identified in *P. aeruginosa*, *A. baumannii* and *Enterobacter cloacae* complex, respectively. However, no direct correlation was observed between resistance to cefiderocol and acquired carbapenemases, and likely resulted from the addition of different mechanisms beyond the production of a carbapenemase.

Overall, 7/13 (53.8%) patients fulfilled pre-defined criteria for clinical cure. Three patients (23.1%) died. One patient required a switch to long-term antibiotic suppressive treatment after cefiderocol discontinuation.

However, among patients whose strains’ susceptibility profiles were analyzed, cure rate was 6/7 (87.5%) with cefiderocol-susceptible isolates, vs. 0/5 with non-susceptible isolates (*p* < 0.01, Fisher’s exact test).

## 4. Discussion

Our study describes the early use of cefiderocol as salvage treatment against MDR Gram-negative pathogens, during the early access compassionate use program in France. Primary indication was respiratory tract infection due to carbapenemase-producing *P. aeruginosa*. Cure rate was 87.5% with cefiderocol-susceptible isolates confirmed per CLSI guidelines at the National Reference Center, vs. 0% with cefiderocol-resistant or -intermediate isolates (*p* < 0.01).

Carbapenem resistance in Gram-negative bacteria is a major threat worldwide [5], with increased mortality, especially among vulnerable patients [6]. Despite the recent development of various combinations of beta-lactams with beta-lactamase inhibitors, our armentarium against carbapenemase-resistant non-fermenting Gram-negative bacteria remains limited [7]. Carbapenem resistance mechanisms include both transmissible (e.g., carbapenemase production), and intrinsic resistance (e.g., porin loss and efflux pump), as in our study [5,8]. The prevalence of carbapenem-resistant non-fermenters now surpass that of Enterobacterales in many settings, representing a challenge for the management of most severe infections [7].

Previous studies showed a high rate of in vitro susceptibility to cefiderocol, prominently in *P. aeruginosa*, *A. baumannii*, and *S. maltophilia*, as compared with other broad-spectrum antibiotics [1,9].

Furthermore, several multi-national surveillance studies demonstrated that cefiderocol exerts in vitro efficacy against 97% of carbapenem-resistant Enterobacterales, and more specifically against 91.1% of ceftazidime-avibactam resistant strains, using a resistance cefiderocol MIC breakpoint of 4 mg/L [9]. Using the same breakpoint, cefiderocol was also active in vitro against most clinical isolates of MDR *P. aeruginosa* and *A. baumannii* (99.2% and 89.7%, respectively).

However, these promising in vitro data were not confirmed in our study, with 5/16 strains not susceptible to cefiderocol at baseline, when analyzed at the National Reference Center. Those 5 strains were also resistant to all new beta-lactam/beta-lactam inhibitors combinations. It suggests that primary resistance to cefiderocol among MDR *P. aeruginosa* might not be rare. Indeed, elevated cefiderocol MICs have been reported for *S. maltophilia* and *P. aeruginosa* [10].

Therefore, it is of paramount importance to appropriately test cefiderocol susceptibility before prescription, especially when *P. aeruginosa* is involved. MIC determination must be conducted with iron-depleted, cation-adjusted Mueller–Hinton broths (as performed within the National Reference Center in our study) [1]. Disc diffusion might be a suitable alternative despite this method performing poorly with *A. baumannii* [11]. The provisional CLSI breakpoint of 4 mg/L seemed sufficient in our small study, but more data are needed, especially since CLSI, the Food and Drug Administration, and EUCAST breakpoints are different, leading to considerable variability when interpreting cefiderocol antimicrobial susceptibility testing [10,11]. Indeed, in our study, we used the CLSI breakpoints, which seem in line with clinical outcome. On the contrary, the use of EUCAST breakpoints, which is of 2 mg/L, would lead to a discrepancy between microbiological and clinical outcome regarding one patient (n°4), infected with a *P. aeruginosa* strain which would be classified as resistant to cefiderocol, but had a favorable clinical outcome. On the other hand, patient n°7, who was infected with a *P. aeruginosa* strain susceptible to cefiderocol according to CLSI breakpoints, presented with clinical failure. This could be explained by the patient’s numerous comorbidities (including immunosuppressive treatments). Our study is, to the best of our knowledge, the first to provide exhaustive characterization of antimicrobial resistance genes among strains targeted with cefiderocol. Future studies are warranted to identify the relation with cefiderocol resistance.

Nonetheless, real-life clinical data regarding cefiderocol use are scarce. In a recent study, Falcone et al. described 10 critically-ill patients with either bacteremia or ventilator-associated pneumonia caused by carbapenem-resistant *A. baumannii*, *S. maltophilia*, or NDM-producing *K. pneumoniae* treated with cefiderocol. All strains had baseline cefiderocol MIC < 2 mg/L. Cure rate and survival rate at 30 days were 70% and 90%, respectively [12].

These results are in line with our study, if we only consider patients with baseline isolates susceptible to cefiderocol according to CLSI guidelines, with a 6/7 (87.5%) cure rate. Results from the CREDIBLE trial suggest a lower clinical efficacy, around 50%, in a heterogeneous population of patients with various infections caused by carbapenem-resistant gram-negative bacteria [13].

The main limits to our study are the small number of patients and the use of cefiderocol only as salvage therapy.

## 5. Conclusions

Early use of cefiderocol in France primarily targeted respiratory tract infections due to carbapenemase-producing *P. aeruginosa*. Cure rate was 87.5% with cefiderocol-susceptible isolates confirmed per CLSI guidelines at the National Reference Center, vs. 0% with cefiderocol-resistant or -intermediate isolates.

## Figures and Tables

**Table 1:** Patients’ characteristics and sequence type, antimicrobial susceptibility, and resistance determinants of isolated strains (*n* = 12), according to Clinical Laboratory Standards Institute breakpoints (2019 update).

Patient	1	2	3	4	5	6	7		8	9	10	11	12
Strain CNR Reference	O81 A4 (Cephyten 266)	O80 J10 (Cephyten 267)	O81 A1 (Cephyten 268)	O80 H7 (Cephyten 240)	O80 H8 (Cephyten 238)	CNR 212 H7	O80 I4 (Cephyten 255)	O80 I5 (Cephyten 256)	O75 H8 (Cephyten 265)	O81 A2 (Cephyten 269)	O80 I3 (Cephyten 254)	O80 H9 (Cephyten 237)	O80 H10 (Cephyten 239)
Age (year)	54	37	54	72	70	67	31		49	63	35	21	59
Type of Infection	RTI	Vascular	RTI + IAA + Vascular	RTI	RTI	Prosthetic joint infection	RTI		RTI + IAA	RTI	RTI + UTI	BJI + SSTI	RTI
Immunosuppression	Yes	No	No	Yes	Yes	No	Yes		No	Yes	Yes	No	Yes
Septic Shock (SOFA Score)	No (1)	No (9)	No (5)	No (8)	No (5)	No (0)	Yes (9)		Yes (14)	No (0)	Yes (8)	No (4)	No (12)
XDR Isolate that Led to Cefiderocol Treatment	*P. aeruginosa*	*A. baumannii*	*A. baumannii*	*P. aeruginosa*	*P. aeruginosa*	*Enterobacter hormaechei* subsp. *hoffmannii*	*K. pneumoniae*	*P. aeruginosa*	*P. aeruginosa*	*P. aeruginosa*	*P. aeruginosa*	*P. aeruginosa*	*P. aeruginosa*
Concomitant Antibiotic Treatment (Dose Per Day)	-	-	CST (6 MUI) + TGC (UK)	-	-	-	CST (4.5 MUI)		CST (4.5 MUI)	CST (6 MUI) + DOX (200 mg)	CST (15 MUI)	CST (6 MUI)	CST (9 MUI)
Sequence Type	ST-654	Unknown *	Unknown **	ST-357	ST-2613	ST-78	Unknown ***	ST-308	ST-357	ST-233	ST-357	ST-357	ST-2102
Carbapenemase	VIM-4	OXA-23	OXA-23	-	VIM-2	-	OXA-48	NDM-1	VIM-2	OXA-836	-	VIM-2	-
Antimicrobial Susceptibility (MIC)													
Amoxicillin	R	R	R	R	R	R	R	R	R	R	R	R	R
Amoxicillin-clavulanate	R	R	R	R	R	R	R	R	R	R	R	R	R
Ticarcillin	R	R	R	R	R	R	R	R	R	R	R	R	R
Ticarcillin-clavulanate	R	R	R	R	R	R	R	R	R	R	R	R	R
Piperacillin	R	R	R	R	R	R	R	R	R	R	R	R	R
Piperacillin-tazobactam	R	R	R	R	R	R	R	R	R	R	R	R	R
Temocillin	R	R	R	R	R	R	R	R	R	R	R	R	R
Cefotaxime	R (>32)	R (>32)	R (>32)	R (>32)	R (>32)	R (>32)	R (>32)	R (>32)	R (>32)	R (>32)	R (>32)	R (>32)	R (>32)
Ceftazidime	R (>32)	R (>32)	R (>32)	R (>32)	R (>32)	R (>32)	R (16)	R (>32)	R (>32)	R (>32)	R (>32)	R (>32)	R (>32)
Cefepime	R	R	R	R	R	R	R	R	R	R	R	R	R
Aztreonam	R	R	R	R	R	R	R	R	R	R	R	R	R
Imipenem	R (>32)	R (32)	R (32)	2	R (>32)	R (8)	I (2)	R (>32)	R (>32)	R (32)	2	R (>32)	R (32)
Meropenem	R (>32)	R (>32)	R (>32)	R (16)	R (>32)	R (16)	R (2)	R (>32)	R (>32)	R (16)	R (16)	R (>32)	R (>32)
Ertapenem	R (>32)	R (>32)	R (>32)	R (>32)	R (>32)	R (>32)	R (4)	R (>32)	R (>32)	R (32)	R (>32)	R (>32)	R (>32)
Ceftolozane-tazobactam	R (>32)	R (>32)	R (>32)	R (>32)	R (>32)	R (>32)	R (16)	R (>32)	R (>32)	R (>32)	R (>32)	R (>32)	R (>32)
Ceftazidime-avibactam	R (>32)	R (>32)	R (>32)	R (>32)	R (>32)	S (8)	S (≤0.25)	R (>32)	R (>32)	R (>32)	R (>32)	R (>32)	R (>32)
Imipenem-relebactam	R (>32)	R (32)	R (32)	2	R (>32)	S (1)	S (1)	R (>32)	R (>32)	R (32)	2	R (>32)	R (16)
Meropenem-vaborbactam	R (>32)	R (>32)	R (>32)	R (16)	R (>32)	I (8)	S (2)	R (>32)	R (>32)	R (16)	R (16)	R (>32)	R (>32)
Cefepime-zidebactam	8	32	32	≤4	4	8	≤4	8	≤4	8	8	8	32
Cefiderocol	S (2)	S (1)	S (0.5)	S (4)	S (2)	S (1)	S (0.5)	S (4)	I (8)	R (16)	R (16)	R (>32)	R (16)
Amikacin	R (64)	S (16)	R (>256)	R (>256)	R (>256)	S (16)	S (4)	R (>256)	R (>256)	R (>256)	R (>256)	R (>256)	S (16)
Gentamicin	R (>256)	R (>256)	R (>256)	R (>256)	R (>256)	R (>256)	S (0.5)	R (>256)	R (16)	I (8)	R (>256)	R (>256)	S (3)
Tobramycin	R (>256)	S (3)	R (>256)	R (>256)	R (>256)	R (48)	S (6)	R (>256)	R (32)	R (>256)	R (>256)	R (>256)	S (1)
Ciprofloxacin	R (>32)	R (>32)	R (>32)	R (>32)	R (>32)	R (>32)	R (1.5)	R (>32)	R (>32)	R (>32)	R (>32)	R (>32)	R (4)
Levofloxacin	R (>32)	R (24)	R (>32)	R (>32)	R (>32)	R (>32)	S (0.5)	R (>32)	R (>32)	R (>32)	R (>32)	R (>32)	R (>32)
Chloramphenicol	R (>256)	R (>256)	R (>256)	R (>256)	R (>256)	R (>256)	S (2)	R (>256)	R (>256)	R (>256)	R (>256)	R (>256)	R (>256)
Colisitin	S (2)	S (2)	S (1)	R (4)	S (2)	S (0.5)	S (1)	S (2)	S (2)	R (64)	S (2)	S (2)	S (2)
Tygecyclin	R (16)	2	4	R (8)	R (16)	S (1)	2	R (8)	R (8)	R (8)	R (8)	R (8)	R (16)
Eravacyclin	R (8)	0.5	1	R (4)	R (8)	S (2)	0.5	R (4)	R (4)	R (4)	R (4)	R (4)	R (8)
Antimicrobial resistance genes													
β-lactams	*bla*_VIM-4_	*bla*_OXA-23_	*bla*_OXA-23_	*bla*_LCR-1_	*bla*_VIM-2_	*bla*_CTX-M-15_	*bla*_OXA-48_	*bla*_NDM-1_	*bla*_VIM-2_	*bla*_OXA-836_	*bla*_LCR-1_	*bla*_VIM-2_	*bla*_PDC-5_
	*bla*_PDC-418_	*bla*_ADC-25-like_	*bla*_ADC-25-like_	*bla*_PDC-223_	*bla*_OXA-415_	*bla*_TEM-1B_	*bla*_CTX-M-15_	*bla*_PDC-19a_	*bla*_OXA-520_	*bla*_PDC-418_	*bla*_PDC-223_	*bla*_LCR-1_	*bla*_OXA-50_
	*bla*_PDC-3_	*bla*_PDC-418_	*bla*_PDC-418_	*bla*_OXA-846_	*bla*_PDC-223_	*bla*_OXA-1_	*bla*_OXA-1_	*bla*_PDC-223_	*bla*_PDC-223_	*bla*_PDC-3_	*bla*_OXA-846_	*bla*_OXA-520_	*bla*_PDC-418_
	*bla*_OXA-396_	*bla*_OXA-66_	*bla*_OXA-66_	*bla*_PDC-373_	*bla*_PDC-35_	*bla*_ACT-24_	*bla*_TEM-1B_	*bla*_OXA-488_	*bla*_OXA-846_	*bla*_OXA-836_	*bla*_PDC-373_	*bla*_PDC-223_	
		*bla*_OXA-396_	*bla*_OXA-396_		*bla*_OXA-488_		*bla*_SHV-11_		*bla*_PDC-11_	*bla*_OXA-486_		*bla*_OXA-846_	
		*bla*_ADC-30_	*bla*_ADC-73_									*bla*_PDC-11_	
Aminoglycosides	*aph(3′)-IIb*	*aph(3′)-Ic*	*aph(3′)-Ic*	*aph(3′)-IIb*	*aph(3′)-IIb*	*aac-(3)-IIa*	*aph(6)-Id*	*rmtD2*	*aph(3′)-IIb*	*aph(3′)-IIb*	*aph(3′)-IIb*	*aph(3′)-IIb*	*aph(3′)-IIb*
	*ant(2″)-Ia*	*aph(3′)-Ia*	*aph(3′)-Ia*	*aac(6′)-Ib*	*aac(6′)-Ib4*	*aac-(6′)-1b-cr*	*aph(3′)-Ia*	*aph(3′)-IIb*	*aac(6′)-Ib*	*aac(6′)-II*	*aac(6′)-Ib*	*aph(3′)-Ia*	
	*aph(6)-Id*	*aph(3″)-Ib*	*aph(3″)-Ib*	*ant(2″)-Ia*	*aac(6′)-II*	aad1	*aph(3″)-Ib*	*aac(3)-I*			*ant(2′′)-Ia*	*aph(3′)-Ib*	
	*aph(3″)-Ib*	*ant(3″)-IIa*	*armA*		*aacA8*	*aph(3″)-IIb*	*aac(6′)-Ib-cr*	*aac(6′)-II*				*aph(6)-Id*	
	*aacA56*	*aac(3)-Ia*										*ant(2″)-Ia*	
Quinolones	mutated *gyrA*			*crpP*	*crpP*	*aac-(6′)-1b-cr*	*aac(6′)-Ib-cr*	*qnrVC1*	*crpP*	*qnrS2*	*crpP*	*crpP*	
				mutated *gyrA*		*qnrB1*	*oqxA*	*crpP*	mutated *gyrA*	*crpP*	mutated *gyrA*	mutated *gyrA*	
							*qnrB1*			mutated *gyrA*			
Sulphonamides	*sul1*	*sul1, sul2*		*sul1*	*sul1*	*sul2*	*sul2*	*sul1*	*sul1*	*sul1*	*sul1*	*sul1*	
Trimethoprim	*dfrB5*					*dfrA14*	*dfrA14*	*dfrB5*					
Tetracyclin	*tet(A), tetR*		*tet(B)*			*tet(A)*	*tet(A)*	*tet(G)*					
Phenicols	*catB7*	*catB7*		*catB7*	*catB7*		*catB3*	*catB7*	*catB7*	*catB7*	*catB7*	*catB7*	*catB7*
Fosfomycin	*fosA*	*fosA*		*fosA*	*fosA*		*fosA*	*fosA*	*fosA*	*fosA*	*fosA*	*fosA*	*fosA*
Polymyxin										mutated *pmrB*			
Macrolides			*mph(E), msr(E)*										
Outcome	Cure	Cure	Cure	Cure	Cure	Cure	Failure		Death (infection)	Death (infection)	Failure	Failure	Suppressive treatment

* 2-185-1-97-3-2-3; ** 2-185-1-96-3-2-3; *** New *gapA* allele-1-2-1-12-1-121; Patients with non-susceptible strains are patients 8 to 12. BJI: bone and joint infection; CNR: National Reference Center for Antimicrobial Resistance; CST: colistin; DOX: doxycyclin; IAA: intraabdominal infection; MIC: minimal inhibitory concentration; RTI: respiratory tract infection; SSTI: skin and soft tissue infection; SOFA: sequential organ failure assessment; TGC: tygecyclin; UK: unknown; UTI: urinary tract infection; XDR: extensively drug-resistant; R: resistant; I: intermediate: S: susceptible.

## Data Availability

All data are available upon request to the corresponding author.
